# The prevalence of rheumatoid arthritis in Spain

**DOI:** 10.1038/s41598-020-76511-6

**Published:** 2020-12-09

**Authors:** Lucía Silva-Fernández, Cristina Macía-Villa, Daniel Seoane-Mato, Raúl Cortés-Verdú, Antonio Romero-Pérez, Víctor Quevedo-Vila, Dolores Fábregas-Canales, Fred Antón-Pagés, Gustavo Añez, Anahy Brandy, Cristina Martínez-Dubois, Paula Rubio-Muñoz, Carlos Sánchez-Piedra, Federico Díaz-González, Sagrario Bustabad-Reyes

**Affiliations:** 1 Rheumatology Department, Complexo Hospitalario Universitario de A Coruña, As Xubias 84, 15006 A Coruña, Spain; 2 Rheumatology, Hospital Universitario Severo Ochoa, Madrid, Spain; 3 Research Unit, Spanish Society of Rheumatology, Madrid, Spain; 4 Rheumatology, Hospital General de Ontinyent, Ontinyent, Valencia Spain; 5 Rheumatology, Complejo Hospitalario de Jaén, Jaén, Spain; 6 Rheumatology, Hospital Comarcal Monforte de Lemos, Monforte de Lemos, Lugo Spain; 7 Rheumatology, Hospital de Barbastro, Barbastro, Huesca Spain; 8 Rheumatology, Complejo Asistencial Universitario de Palencia, Palencia, Spain; 9 Rheumatology, Hospital Universitario Puerta del Mar, Cádiz, Spain; 10 Rheumatology, Hospital Universitario Central de Asturias, Oviedo, Asturias Spain; 11 Rheumatology, Hospital Universitario Marqués de Valdecilla, Santander, Cantabria Spain; 12 Rheumatology, Hospital Universitari Germans Trias I Pujol, Badalona, Barcelona Spain; 13 Universidad de La Laguna, La Laguna, Santa Cruz de Tenerife Spain; 14 Rheumatology, Hospital Universitario de Canarias, La Laguna, Santa Cruz de Tenerife Spain

**Keywords:** Rheumatoid arthritis, Epidemiology, Population screening

## Abstract

Rheumatoid arthritis (RA) prevalence is believed to be around 1% worldwide, although it varies considerably among different populations. The aim of EPISER2016 study was to estimate the prevalence of RA in the general adult population in Spain. We designed a population-based cross-sectional study. A national survey was conducted between November 2016 and October 2017 involving a probabilistic sample from the general population aged 20 years or older. Subjects were randomly selected for phone screening using a computer-assisted telephone interviewer system. Positive RA screening results were evaluated by a rheumatologist. Cases fulfilled the 1987 ACR and/or the 2010 ACR/EULAR criteria; previous diagnosis established by a rheumatologist and clearly identified in medical records were also accepted regardless of the criteria used. Prevalence estimates with 95% CI were calculated taking into account the design of the sample (weighting based on age, sex, and geographic origin using as a reference the distribution of the population in Spain). 4916 subjects participated in the study and 39 RA cases were confirmed. RA estimated prevalence was 0.82% (95% CI 0.59–1.15). Mean age of RA cases was 60.48 (14.85) years, they were more frequently women (61.5%), from urban areas (74.4%), non-smokers (43.6%), and with a high body mass index (53.8% with overweight). Extrapolating to the population in Spain (approximately 37 million are ≥ 20 years old), it was estimated that there were between 220,000 and 430,000 people aged 20 years or older with RA. No undiagnosed cases were detected, which could be related to the establishment of early arthritis clinics around the country, increasing the rates of diagnosis during early phases of RA.

## Introduction

Rheumatoid arthritis (RA) is the most common chronic inflammatory rheumatic disease^1^. Its prevalence is believed to be around 1% worldwide, although it varies considerably among different populations^2^. Studies conducted in North America have shown prevalences of 0.5–1.1%^3–5^. In Europe, there seems to be a latitude gradient with higher prevalences in the north. Indeed, studies from Germany^6^ and Sweden^7^ found a prevalence around 0.65%, whereas in other European countries like France^8^ or Italy^9^, the prevalence was lower than the global estimation and ranged between 0.19 and 0.41%. Age and sex differences add more variability to RA prevalence. Several studies^10,11^ have reported the prevalence in men to be between 0.09 and 0.16%, while in women it can reach 1.54%^12^. The effects of several environmental factors such as smoking, diet and/or certain infections on the risk of RA are well known and differ between populations^13^. Further variation occurs as a result of differences in statistical methods and case-ascertainment criteria.

In Spain, the EPISER study (a national survey conducted in 2000) estimated that the prevalence of RA in the adult population was 0.5% (95% confidence interval [CI] 0.25–0.85%). RA prevalence was higher in women (0.8%) than in men (0.2%). Taking into account the Spanish population at that time, it was thought that there were between 150,000 and 200,000 people over 20 years of age with RA in the entire country^14^.

Socioeconomic, demographic and lifestyle behaviour changes over the last 16 years may have influenced the prevalence and the characteristics of RA in Spain. On the other hand, knowledge about RA has grown exponentially, both in terms of early diagnosis and treatment. It is possible that after the publication of the 2010 ACR/EULAR classification criteria, which identify RA patients during earlier phases of the disease, the estimated prevalence of RA has increased, as it has been observed in other settings^15^. Besides, the proliferation of early arthritis clinics has contributed not only to earlier diagnoses, but also to improvements in prognosis^16^. More recent treatment strategies like “treat-to-target” and the development of new drugs have also contributed to increased survival rates^17^.

The aim of EPISER2016, promoted by the Spanish Society of Rheumatology, was to update the estimated prevalence of RA in Spain and, as a secondary objective, to evaluate its association with sociodemographic, anthropometric and lifestyle variables.

## Methods

### Study design and sample selection

The methods and general characteristics of the sample have been previously published^18,19^. In brief, EPISER2016 was a population-based cross-sectional study, based on a national survey and conducted between November 2016 and October 2017. The main purpose of the study was to estimate the prevalence of RA, ankylosing spondylitis, psoriatic arthritis, gout, systemic lupus erythematosus, Sjögren syndrome, symptomatic osteoarthritis of cervical and lumbar spine, hands, hips and knees, fibromyalgia, and symptomatic osteoporotic fracture. This analysis only included RA.

Multistage stratified cluster random sampling was used: first, by autonomous communities (regions into which Spain is administratively divided), then by rural/urban character of the municipalities (i.e., urban if at least one town in the municipality with ≥ 10,000 inhabitants) and finally, by sex and age (10-year-wide) strata. EPISER2016 included a probabilistic sample of individuals from the general population aged ≥ 20 years who were residents in one of the randomly selected municipalities and who were capable of communicating with the interviewers. For 3 of the 17 autonomous communities the randomized municipalities were not representative, mainly because of their sociodemographic characteristics such as a higher percentage of foreigners or second homes, so they were replaced by another randomly selected municipality in the same autonomous community.

### Recruitment, data collection and RA screening

Subjects were randomly selected for phone screening using a computer-assisted telephone interviewer (CATI) system. The survey was mostly performed via landlines, but in order to facilitate access to younger patients and expand the registry, we incorporated mobile phones since March 2017, representing 20.3% of the final sample. This figure reflects the proportion of homes in Spain that relied solely on a mobile telephone connection (19). In case of non-answered phone calls, a minimum of six attempts were made in different time frames. If after these attempts there was no answer or the subject refused to participate, another phone number within the same municipality was randomly selected. Both for the randomized selection of telephones in each municipality as well as for conducting the initial screening interviews, an external company working in sociological studies was involved, with experience in the area of health and call center services (Ipsos España).

The screening considered two complementary paths for all the participants. First, they were asked if they had already been diagnosed with RA. If a participant reported such a diagnosis, his/her consent was sought to allow the rheumatologists who were participating as researchers at the municipality’s referral hospital to review the presence of this diagnosis in their clinical records.

**Figure 1:**
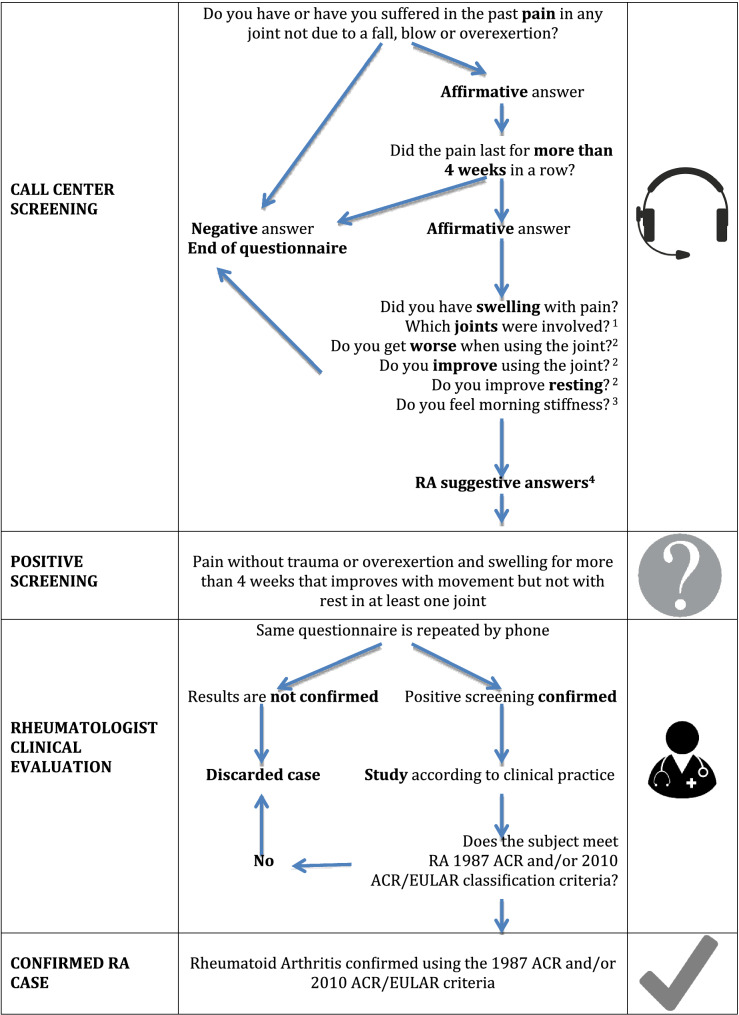
Symptom-based screening flowchart in EPISER2016 for individuals not previously diagnosed with Rheumatoid Arthritis. *RA* rheumatoid arthritis. ^1^Subject was questioned about pain with swelling that lasted more than 4 consecutive weeks in a row without any fall, blow or trauma, particularly in the hands, hips/groins, knees, ankles and feet, specifying if location was on the left side, right side or both. ^2^These questions were referred only to the areas previously referred to as painful. ^3^In the case of an affirmative answer, there was an additional question consulting if duration was greater than 30 min. ^4^Considered suggestive of RA: an affirmative answer to pain improvement with activity, plus a negative answer to improvement with rest.

Subjects also answered a generic arthritis symptoms questionnaire, involving a symptom-based screening method summarized in Fig. 1. A subject was classified as "positive RA screening" if he/she reported to have or have had pain and swelling without trauma or overexertion in a joint for more than four weeks in a row, which improved with movement but not with rest. These "positive RA symptom-based screening" cases were forwarded to a pre-assigned rheumatologist (at least one in each geographical area encompassed by the study), who performed a second phone interview if the subject was not previously diagnosed. If disease was suspected in this second interview, the rheumatologist performed a face-to-face evaluation of the patient and requested all relevant tests to confirm if the subject had RA, based on the 1987 ACR criteria^20^ for RA and/or the 2010 ACR/EULAR criteria^21^.

All patients with a previous diagnosis of RA established by a rheumatologist (clearly identified in their medical records regardless of the criteria used) and those confirmed by the rheumatologist after the face-to-face evaluation were classified as RA cases.

### Ethics

EPISER2016 was developed according to the protocol and good clinical practice standards of the International Conference on Harmonization. Oral informed consent was obtained from all subjects during the first phone contact. Their agreement or refusal was recorded. Written informed consent was obtained from all subjects seen in clinics according to the Declaration of Helsinki, authorizing physical examinations and complementary tests if needed. The study was initially approved by the Research Ethics Committee of the Hospital Universitario de Canarias (Acta 12/2016) and subsequently by the ethics committees of all the participant hospitals that requested it.

### Sample size

Assuming a Poisson distribution, a sample comprising 4000 individuals would enable to obtain a 95% CI of 0.30–0.77 for an expected RA prevalence of 0.5%. Subjects who completed the call center interview with a positive result for the screening of RA and the rheumatologist could not access their clinical records and/or contact them to confirm or rule out the diagnosis were considered missing. Assuming 20% of missing values, it was deemed necessary to include around 5,000 individuals (18, 19).

### Statistical analysis

Prevalence and the 95% CI were calculated, taking into account the sample design. Weighting was applied depending on the probability of selection at each of the sampling stages, using as a reference the distribution of the population in Spain in 2016 according to Continuous Register Statistics from the Spanish Institute of Statistics (https://www.ine.es/en/index.htm). Weights were calculated based on age (10-year strata), sex, and geographic origin (three areas were defined: North [Galicia, Asturias, Cantabria, Basque Country, Navarra and La Rioja], Mediterranean and Canary Islands [Catalonia, Comunidad Valenciana, Balearic Islands, Murcia, Andalusia and Canary Islands], and Central [Comunidad de Madrid, Castilla y León, Aragón, Castilla-La Mancha and Extremadura]); according to these characteristics each individual in the sample represented a certain number of people from the general population^19^.

Lastly, models were constructed to analyse which of the sociodemographic, anthropometric, and lifestyle variables included in the call centre questionnaire were associated with RA. First, a bivariable analysis of the association between RA and each of the variables was performed. Then, multivariable binary logistic regression models were constructed based on those variables with a p value < 0.2 in the bivariable analysis (age and sex were included in the model regardless of the p value in the bivariable analysis). Statistical significance was defined as p value < 0.05.

Due to feasibility issues, the validity of the screening questionnaire could not be evaluated prior to the study. Nevertheless, we calculated the positive predictive value (PPV) of the questionnaire taking into account the cases confirmed subsequently. In addition, we carried out a substudy of the negative predictive value (NPV) in a sample of 209 subjects randomly selected from those who had a negative screening result for all of the diseases studied in EPISER2016. These subjects were contacted by telephone by the research rheumatologist in their catchment area with the aim of confirming the negative result. If it was not possible to rule out a positive result in the phone call, the patient was attended for examination and performance of the relevant additional tests according to the disease suspected (18, 19).

SPSS statistical package version 22 (SPSS Inc, Chicago, IL) was used for the statistical analysis.

## Results

A total of 84,098 different phone numbers were dialed. Of these, 50,170 were wrong numbers or were unanswered; 28,784 individuals refused to participate (27,895 or 96.9% from the very beginning of the interview) and 5144 interviews were completed. The response rate, once the individual had been contacted, was 15.2% (19).

After removing duplicate interviews or excess numbers from certain sample strata, a total of 4916 subjects from 78 municipalities distributed across the 17 Spanish autonomous communities were included in the analysis. 54.5% of the subjects were women and 67.9% were ≥ 40 years old. 77.5% were residents in urban municipalities. These percentages are in accordance with the characteristics of the adult population in Spain; the greatest difference was in the percentage of people born abroad, with a lower proportion in the sample than in the general population (7% and 14.5%, respectively). A detailed description of the sample and a comparison with the general population aged 20 years or older in Spain (reference population in EPISER2016) has been published elsewhere^19^.

### Predictive value of the questionnaire for RA screening

Of the 4916 subjects interviewed, 289 (5.9%) had a positive RA screening result. Of these, 7 (2.4%) were missing subjects. Following the rheumatologists' assessments, 39 RA cases were confirmed without need of clinical visits or complementary tests, since all of these patients had a previous established RA diagnosis. Of them, 36 were based on the 1987 ACR and/or the 2010 ACR/EULAR criteria. The other three were clinician-based diagnoses; two of them did not fulfill ACR criteria and for the other one there was not enough information to confirm it.

The PPV of the RA screening questionnaire was 12.77% (36 confirmed cases among the 282 subjects with positive screening who had completed the study). The calculation was made with 36 cases instead of 39 because 3 of them had a positive result in the screening for one of the other diseases included in EPISER2016, but not for RA.

The NPV for RA of the complete screening questionnaire was 100% (0 confirmed cases among 209 subjects without a positive screening result for any of the diseases included in EPISER2016 who had been randomly selected for the NPV study).

### Prevalence of RA in Spain in 2016

The estimated RA prevalence in Spain in 2016 was 0.82% (95% CI 0.59–1.15). After extrapolating to the total population of the country (approximately 47 million, of which 37 million are ≥ 20 years old), it was estimated that in 2016 there were between 220,000 and 430,000 people aged 20 years old or over with RA in Spain. Considering only the 33 cases that fulfilled the 1987 ACR criteria, the prevalence was 0.69% (95% CI 0.47–0.99).

### Characteristics of RA cases and disease-associated variables

The mean age was 60.48 years (SD 14.85). Twenty cases (51.3%) were 60 years old or over. RA cases were more frequently women (24 cases; 61.5%). With respect to education levels, 22 (56.4%) had attained a basic education. In terms of smoking, 17 (43.6%) patients had never smoked, 12 (30.8%) were ex-smokers, and 10 (25.6%) were current smokers. Most of the cases had overweight (53.8%).

Table 1 shows the results of the bivariable analysis for the association between the presence of RA and sociodemographic variables, physical characteristics and smoking. Table 2 presents the multivariable analysis results. Age over 60 years was significantly associated with the presence of RA (OR 6.436; 95% CI 1.775–23.345; p = 0.005). Interestingly, RA prevalence was lower in subjects with higher education levels, although this trend needs additional confirmation. Current smoking was associated with RA, although statistical significance was not reached.

**Table 1:** Presence of rheumatoid arthritis in relation to sociodemographic, physical and lifestyle variables.

Variable	RA cases (39)	Subjects without RA (4870)	p
**Age (%)**			0.001
20–39	7.7	32.4	
40–59	41.0	38.4	
≥ 60	51.3	29.2	
Sex (females) (%)	61.5	54.4	0.370
**Geographic area (%)**			0.643
North	23.1	28.8	
Mediterranean and Canary Islands	48.7	41.9	
Centre	28.2	29.3	
**Educational level (%)**			0.033
Basic	56.4	37.1	
Medium	23.1	26.0	
High	20.5	36.9	
**BMI (%)**			0.246
Normal weight (18.5 ≤ BMI < 25)	38.5	44.5	
Underweight (BMI ≤ 18.5)	0	1.2	
Overweight (25 ≤ BMI < 30)	53.8	39.4	
Obesity (BMI ≥ 30)	7.7	14.9	
**Smoking habit (%)**			0.772
Never smoker	43.6	49.2	
Former smoker	30.8	26.8	
Current smoker	25.6	24.1	
Born abroad (%)	7.7	7	0.751
Residence in an urban municipality (%)	74.4	77.5	0.638

Bivariate analysis.

*BMI* body mass index.

**Table 2:** Multivariable analysis.

Variable	OR	95% CI	p
**Age**			
20–39	Ref		
40–59	4.099	1.156–14.533	0.029
≥ 60	6.436	1.775–23.345	0.005
**Sex**			
Male	Ref		
Female	1.020	0.515–2.018	0.955
**Educational level**			
Basic level	Ref		
Medium level	0.782	0.348–1.756	0.551
High level	0.566	0.241–1.333	0.193
**Smoking habit**			
Never	Ref		
Former smoker	1.266	0.583–2.752	0.551
Current smoker	1.588	0.694–3.635	0.274

Variables associated with the presence of rheumatoid arthritis.

*OR* odds ratio, *CI* confidence interval.

### Comparison with EPISER2000

Table 3 summarizes a comparison between EPISER2000 and EPISER2016. The latter sample was 2.2 times bigger than that in EPISER2000. The 2016 study also incorporated the new 2010 ACR/EULAR RA classification criteria, which are more sensitive for early stages of the disease. The PPV of the initial questionnaire was improved considerably, while maintaining the NPV at 100%. It must be taken into account that while the EPISER2016 NPV was calculated based on a subsample of 209 subjects, in EPISER2000 a rheumatologist evaluated all of the subjects. EPISER2000 built its sample via postal mailings, while EPISER2016 used phone calls.

**Table 3:** Comparison between EPISER2000 and EPISER2016 studies.

	EPISER2000	EPISER2016
Sample, n	2192	4916
RA classification criteria used	1987 ACR	1987 ACR, 2010 ACR/EULAR and previous diagnosis by a rheumatologist
Positive screening result^a^, n (%)	186 (8)	289 (5.9)
Confirmed RA cases, n	11	39
Screening questionnaire PPV (%)	5.9	12.77
Screening questionnaire NPV (%)	100	100
RA prevalence based on ACR 1987 criteria, % (95% CI)	0.5 (0.3–0.9)	0.69 (0.47–0.99)
Estimation of total RA cases in Spain^b^, n	80,000–271,000	220,000–430,000
Diagnosis of RA prior to EPISER study, n (%)	8 (72.7)	39 (100)
**Sociodemographic characteristics**		
Females, n (%)	9 (81.8)	24 (61.5)
Prevalence in females, % (95% CI)	0.8 (0.4–1.3)	0.9 (0.6–1.3)
Prevalence in males, % (95% CI)	0.2 (0.0–0.5)	0.8 (0.4–1.3)
Mean age, years ± SD	59.27 ± 22.01	60.48 ± 14.85
Residence (%)		
Urban	–	74.4
Rural	–	25.6
Educational level (%)		
Basic	63.6	56.4
Medium	18.2	23.1
High	18.2	20.5

*RA* rheumatoid arthritis, *SD* standard deviation, *CI* confidence interval, *PPV* positive predictive value, *NPV* negative predictive value.

^a^Screening questionnaire: EPISER2000 used a questionnaire administered by a rheumatologist in a face-to-face interview, while EPISER2016 used a phone questionnaire administered by the call centre interviewers.

^b^These estimates were calculated considering the lower and upper limits of the confidence intervals of the prevalence and the figures of the population aged 20 years old or over in Spain in the years 2000 and 2016. The confidence interval used for EPISER2016 was that corresponding to the prevalence of 0.82%.

Considering only the cases that fulfilled the 1987 ACR criteria, the RA prevalence in Spain based on EPISER2016 was higher than in EPISER2000 (0.69 and 0.5, respectively), although the confidence intervals overlap. As with EPISER2000, EPISER2016 identified an association between RA and age. EPISER2000 did not collect data on smoking or BMI.

## Discussion

The burden of chronic diseases like RA is growing. Given the increasing treatment costs of the disease, information on the frequency of RA is crucial in predicting the socioeconomic impact of the disease and developing specific plans for health-care systems^22^. In 2000, the Spanish Society of Rheumatology (SER) conducted the first nation-wide survey to estimate the prevalence of several rheumatic diseases^23^. Sixteen years later, sociodemographic and lifestyle changes such as the ageing of the population or smoking habits justified a new study to establish the influence of these changes in the prevalence of rheumatic diseases. EPISER2016 estimated the prevalence of RA in Spain at 0.82% (95% CI 0.59–1.15).

This finding is in agreement with other studies, mainly from northern Europe^6,7,24^, and is higher than the prevalence found in southern countries^8,9^. This difference with the surrounding countries may stem from the different methodologies and the varied case definitions employed. The majority of the prevalence estimations are based on national health databases^6,9,11^. Nevertheless, studies with methodologies similar to EPISER2016 have found lower RA prevalences. A Serbian study, following a methodology proposed by an European EULAR project, used an approach similar to our own^10^. They also included two phases (a detection phase consisting of a telephone questionnaire led by trained interviewers and a confirmation phase overseen by a rheumatologist). The case definition required a rheumatologist-made clinical diagnosis, with the age- and sex-standardized prevalence measuring 0.35% (0.16% in men and 0.51 in women). Comparisons with EPISER2016 can be partly affected by certain differences in the sampling procedures and statistical analyses. Another study carried out in the southern part of Denmark^25^, which also included an initial mail questionnaire in conjunction with a telephone interview and the clinical examination, established a point prevalence of RA, based on the 1987 ACR criteria, of 0.35% (95% CI 0.17–0.52) while the prevalence of RA in Spain using these same criteria was 0.69% (95% CI 0.47–0.99). The cumulative prevalence in the Danish study was 0.92% (95% CI 0.62–1.21). The authors acknowledge that the point prevalence was most likely underestimated. Additionally, in this study individuals over the age of 15 were included, which may have influenced the global estimation due to the low prevalence of RA in this age bracket. The different genetic and environmental backgrounds of the populations would explain the different results between EPISER2016 and studies with similar methodologies.

With regard to the changes in RA within Spain during this sixteen-year period, although larger sample sizes are needed to calculate more precise estimations, there seems to have been an increase in the frequency of the disease. This would be in line with the results of a recent systematic analysis of the Global Burden of Disease study, which has shown an increase in age-standardised prevalence from 1990 to 2017 in different regions of the world, including Western Europe^26^.

EPISER2016 was designed to estimate the global prevalence of several rheumatic diseases in Spain. The analysis of the association with sociodemographic, anthropometric and lifestyle variables was a secondary objective and for pathologies with a low prevalence, such as rheumatoid arthritis, the statistical power for this analysis is very limited. Taking this into account when interpreting the data, we did not observe a significant difference between men and women. Nevertheless, the upper limit of the 95% CI in the multivariate analysis (around twice as prevalent in women than men) would be in line with ratios reported in previous studies, such as in the USA and Canada^3,27,28^. The mean age of the cases was similar to that of EPISER2000^14^ and again, both in the bivariable and multivariable analysis, age was associated with the presence of RA.

Unlike the previous study, EPISER2016 did not identify any RA patient not previously diagnosed (EPISER2000 found three cases undiagnosed among 11). The establishment of early arthritis clinics (EACs) around the country has probably reduced in a substantial way the waiting time for arthritis patients to be seen by a rheumatologist, subsequently increasing the rates of diagnosis during early phases of RA. A number of studies^29–31^ have reported an important reduction in the referral time to a rheumatologist following the establishment of an EAC, one of them reporting an impressive reduction in average referral time from 10 weeks to 1 day ^32^.

One of the strengths of our study comes from the sample included, which can be considered representative of the population aged 20 years or over in Spain for the aims of this study, and with an adequate sample size to reliably estimate the prevalence of RA^19^. The difference in the percentage of people born abroad, with a lower proportion in the sample than in the general adult population (7% and 14.5%, respectively), could be explained by the difficulty of access to the population of certain nationalities, due to differences in language. Nevertheless, the influence of race/ethnicity on the prevalence of RA would be limited^28,33^, so it would not invalidate the results obtained. In addition, since all of the suspected RA cases were confirmed by a rheumatologist, we are confident that our method of case ascertainment avoided both under- and overestimations.

Prevalence studies usually include two phases: the screening phase (in this case by telephone interview) and the confirmation phase. A telephone interview that relies only on landline phone numbers has its own limitations, mainly due to the fact that younger people usually only have a mobile phone; it is those aged 50 years or older who will likely answer a landline^34^. To minimize this bias, we also used mobile-phone databases in 20% of the interviews, a figure similar to the proportion of homes in Spain relying solely on a mobile telephone^19^. An under- or overestimation of the actual prevalence could arise from this detection phase if for any reasons a high number of RA patients were prone or reluctant to participate during the detection phase. In this sense, there was no statistically significant difference in the percentage of positive RA screening results between the 294 subjects who completed the initial screening phase by the call center, but refused to give their consent to participate in the confirmation phase, and the 4916 subjects who agreed to participate in this confirmation phase (8.16% vs 5.88%; p = 0.109).

Telephone surveys have become an accepted method for prevalence studies on rheumatic diseases^10,35,36^. The response rate for the initial screening phone calls was 15.2%, which represents a possible source of bias. Nevertheless, this rate is in accordance with other recent telephone surveys and could have been lower compared to other studies because of higher sampling requirements (strata based on sex, age and population of the municipality)^37,38^. According to different reports, a low response rate is not necessarily a source of bias. It will depend on the reasons for non-participation. If there is no relation to the important variables of the study, there may be no bias^37,39–41^. In this respect, the 2017 National Health Survey of Spain, which used meticulous sampling procedures, found similar frequencies of self-reported osteoarthritis, chronic cervical pain and chronic lumbar to those reported by EPISER2016 participants (20,6% vs 18,4%; 17,4% vs 13,5%; 21,7% vs 18,4%, respectively)^19,42^. This means that the participation rate in EPISER2016 is not probably a source of bias in our investigation, given that the reasons to refuse participation are most likely unrelated to the main aim of the study.

We initially considered other alternatives, but phone calls came up as the most suitable option in our environment to access the general population. In Spain, no administrative claims are available to estimate the prevalence of RA for the entire country. In the case of other recruitment alternatives, i.e. postal mail, they required more complicated logistics and we had no guarantee of a better response.

## Conclusions

The prevalence of RA in the general population aged 20 years or over in Spain appears to have increased compared to the rate reported in 2000, although larger sample sizes would be needed to confirm this. In total, we estimate that there were between 220,000 and 430,000 persons with RA in Spain in 2016. The prevalence is higher in individuals aged 60 years or older and there seems to be a trend towards a lower prevalence in those with a higher level of education, although this trend needs additional confirmation. No undiagnosed cases were detected. Studies like EPISER2016 are crucial to understanding the socioeconomic impact of the rheumatic diseases and to guiding future public health policies.

## Data Availability

The dataset generated and analysed during the current study is available from the corresponding author or from Spanish Foundation of Rheumatology (proyectos@ser.es) on reasonable request.
